# Knowledge and Recommendations of Stakeholders Regarding Ethical Oversight of Data Science Health Research: Protocol for a Qualitative Study

**DOI:** 10.2196/78557

**Published:** 2025-12-18

**Authors:** Clement Adebamowo, Adeola Akintola, Oluchi C Maduka, Peter Ikhane, Ayodele Jegede, Shawneequa Callier, Simisola Akintola, Temidayo Ogundiran, Olusegun Adeyemo, Sally N Adebamowo, BridgELSI Project as part of the DS-I Africa Consortium

**Affiliations:** 1 Department of Epidemiology and Public Health University of Maryland School of Medicine Baltimore, MD United States; 2 Department of Research Center for Bioethics and Research Ibadan Nigeria; 3 University of Maryland Marlene and Stewart Greenebaum Comprehensive Cancer Center Baltimore, MD United States; 4 Department of Private and Property Law University of Ibadan Ibadan Nigeria; 5 Department of Bioethics and Medical Humanities University of Ibadan Ibadan Nigeria; 6 Department of Sociology University of Ibadan Ibadan Nigeria; 7 Department of Clinical Research and Leadership, School of Medicine and Health Sciences George Washington University Washington DC United States; 8 Center for Research on Genomics and Global Health National Human Genome Research Institute Bethesda, MD United States; 9 Department of Surgery University of Ibadan Ibadan Nigeria; 10 See Acknowledgments

**Keywords:** data science, health research, ethics, ethical oversight, Nigeria, low- and middle-income countries (LMICs), qualitative research, stakeholder engagement

## Abstract

**Background:**

Data science health research (DSHR) uses novel computational methods and high-performance computing to analyze big data from conventional and nonconventional health and related sources to generate novel insights and communications. DSHR creates assets but generates ethical, legal, and social challenges. Key gaps in current ethical oversight of DSHR include blurred boundaries between research and nonresearch data use, inadequate protection of data donors, power imbalances that risk extractive research practices, algorithmic biases, and regulatory inadequacies. Nigeria, a typical low- and middle-income country with rapidly expanding DSHR, exemplifies this environment and concerns.

**Objective:**

This study will elicit answers from Nigerian DSHR stakeholders and contribute to understanding the ethical, legal, and social implications (ELSI) of DSHR and developing novel ethical oversight frameworks.

**Methods:**

Between October 2024 and January 2025, we conducted Key Informant Interviews with 65 stakeholders of 87 individuals. The Key Informant Interview guide comprised 11 construct-based question domains addressing awareness of policies and laws, ethical oversight processes, ELSI considerations in policy development, experiences addressing DSHR challenges, organizational and procedural frameworks, ideal oversight components, stakeholder roles, research impact on ethics and policy, regulatory influences on research practices, equity-enhancing policies, and balanced regulations. The interviews lasted 60-90 minutes and were transcribed. We analyzed the transcripts using a hybrid deductive-inductive approach. A priori codes derived from research objectives provided the analytical framework while allowing for the identification of emergent concepts. The iterative 3-level coding process involved initial code generation, evaluation, and refinement, with codes grouped into thematic families and semantic networks representing hierarchical concept relationships. Query tools and Boolean operators were used to interrogate the codes to extract findings.

**Results:**

Of 87 invited individuals, 22 (25%) were unable to participate. The 65 participants (age: mean 47.9, SD 7.9 years; 50/65, 77% male) included data science health researchers (25/65, 39%), biomedical researchers (17/65, 26%), Health Research Ethics Committee members (12/65, 19%), and policymakers (11/65, 17%). Most held doctoral degrees (38/65, 57%) and were affiliated with academic institutions (45/65, 69%) and government organizations (26/65, 40%), and had received general research ethics training (50/65, 77%). However, only 12% (8/65) had received predominantly short-duration ethics-specific DSHR training, while 92% (60/65) acknowledged the need for specialized DSHR ethics education. As of January 2025, the interview transcripts have been generated, with checking completed, with qualitative analysis scheduled for completion by March 2025 and completion of primary manuscripts by the end of 2025.

**Conclusions:**

This study will generate stakeholder-informed recommendations for ethical oversight of DSHR that address issues relating to broad consent, ELSI, data ownership, benefit-sharing, and donor protection in resource-limited settings. Our findings will inform global DSHR and research ethics communities on the development of contextually appropriate oversight mechanisms that promote equitable partnerships, co-ownership, and tiered data governance.

**International Registered Report Identifier (IRRID):**

DERR1-10.2196/78557

## Introduction

Data science health research (DSHR) is a novel and rapidly growing field derived from several disciplines that coalesced around the use of novel computational methods and high-performance computing to collect, store, manipulate, and analyze huge amounts of health and related data to generate results or reports and novel insights [1]. The sources and types of data used in data science research come from the full spectrum of human activities [2]. They include conventional data from national and institutional health records, various surveillance data, regular systematic and opportunistic surveys, and data from research projects [2-4]. The nonconventional sources of data for data science research include social media, geospatial data, and wearables [5-7]. Linkage of these data sources results in deeper and richer datasets, which, when combined with advanced computational methods such as advanced deep learning, machine learning, and artificial intelligence (AI), create highly valuable assets for data science research, innovation, and discovery [8,9]. The generation, collection, and analyses of these conventional and nonconventional data for health research and discovery poses considerable ethical, legal, and social implications (ELSIs) challenges that require urgent response by research ethicists, regulatory agencies, and policymakers, particularly in low- and middle-income countries (LMICs) such as Nigeria.

Research generating enormous amounts of data that uses data science methods is increasing in LMICs, including Nigeria. In Africa, programs such as the Human Heredity and Health in Africa, Health Education Partnership Initiative, and President’s Emergency Plan for AIDS Relief laid the foundation for DSHR by generating data and developing guidance for research ethics and data sharing based on the classical principles of research ethics. However, DSHR poses unique challenges to these classical ethical principles [10].

DSHR occurs in a pervasive data environment where digital footprints from nonresearch activities (eg, social media and wearables) are often repurposed for health research, sometimes without the awareness or consent of the data participants [11,12]. This blurs the boundaries between traditional health research data and other data types, complicating ethical oversight, particularly when consent modalities for primary data collection are unclear or inadequate [13,14]. Secondary data use and analysis are pervasive in DSHR, and some of these data may have been collected in nonresearch contexts or under outdated consent frameworks. This raises critical questions about confidentiality, data ownership, representational veracity, and privacy. While anonymization and deidentification are standard safeguards in regular research projects, advanced data science methods can reidentify participants through linkage of disparate datasets, thereby undermining assumptions of confidentiality [15-17]. Furthermore, DSHR’s reliance on heterogeneous datasets—collected across diverse contexts, jurisdictions, and timelines—risks accumulating biases and ambiguities, which may distort interpretations and harm communities if results lack contextual fidelity [18,19].

Another issue of concern, particularly in LMICs, is the power imbalance on account of the “inverse care law” and digital data poverty arising from the limited availability of data. There are also challenges with extractive data practices where international researchers or entities leverage infrastructural disparities to conduct “helicopter research” without equitable and empowered inclusion or with token inclusion of local researchers or communities [20,21]. This “predatory inclusion” practice perpetuates data colonization, where international researchers or entities exploit LMICs’ data for profit without fair benefit-sharing, thereby eroding public trust in research in those countries [22,23]. Furthermore, new biases such as algorithmic bias due to a lack of diversity in the data used to build models and among data science researchers further compound these challenges [24,25]. These kinds of biases perpetuate health inequities when the outcome of DSHR is deployed for clinical or public health use without rigorous scientific, methodological, and ethical scrutiny.

In Nigeria, a typical LMIC, the National Code for Health Research Ethics mandates ethics committee review of all research projects, including DSHR, even when secondary data is used [26]. However, there are regulatory gaps with cross-border DSHR projects because the mechanisms for harmonizing ethics reviews across jurisdictions remain underdeveloped [27]. Additionally, pressures from the involvement of commercial entities create new challenges for the traditional ethics review processes, which lack the resources and ability to enforce ethical oversight of research conducted by international, multinational, extra-territorial individuals or corporations and prevent profit-driven data exploitation [22].

Several characteristics of the LMICs environment—poor democratic governance, weak legal infrastructure, high rates of poverty, low levels of education, and poor information technology penetration—lead to limited ability to protect the interests and guarantee of benefits of research to local researchers, research participants, and local communities. This raises significant concerns about the validity and thoroughness of ethical oversight of DSHR in these environments [28,29]. Lack of global rules and enforcement mechanisms remains a major concern that is particularly relevant in DSHR, given the transnational nature of the generation, collection, storage, and manipulation of data across national borders. Most LMICs cannot afford local data centers, given their high cost; hence, their data must be “exported” if they are to contribute to the “big data” used in some DSHR projects.

The ethical challenges caused by DSHR, therefore, demand novel ethical frameworks to address the blurred consent boundaries, reidentification risks, algorithmic biases, and the risk of commercial exploitation, while ensuring equitable representation and benefit-sharing for all populations [30,31]. To ensure this, we are conducting a qualitative study that engages all stakeholders in DSHR with the intention of answering these research questions:

1. What is the current level of awareness and knowledge among stakeholders regarding policies, laws, and ethical frameworks governing DSHR in Nigeria?
2. How effectively do existing ethical oversight mechanisms address the unique ELSI of DSHR in the Nigerian context?
3. What are stakeholders’ experiences with addressing ELSI challenges in DSHR, including issues of consent, privacy, data ownership, and algorithmic bias?
4. What components should constitute an ideal ethical oversight framework for DSHR in Nigeria, considering both international standards and local contexts?
5. How can Nigeria implement adequate ethical regulations while facilitating DSHR, international collaborations, and partnerships that prioritize health research goals and innovations?
6. What roles should various stakeholders, including researchers, patients, participants, communities, policymakers, and ethics committees, play in developing and implementing ethical oversight of DSHR?

The primary outcomes of interest in this study are:

1. Perspectives on broad consent mechanisms appropriate for DSHR contexts, particularly regarding secondary data use, future research applications, and the complexities of obtaining informed consent in pervasive digital data environments.
2. Comprehensive recommendations addressing the ELSIs of DSHR, including strategies for preventing algorithmic bias, protecting data privacy despite reidentification risks, ensuring equitable benefit-sharing, and addressing power imbalances in international research collaborations.
3. Guidance on data ownership structures, benefit-sharing arrangements with data donors and their communities, and protections for primary data donors from potential harms arising from big data analytics and algorithmic biases.

Historically, the development of ethical oversight lags novel research methodologies and technologies; nevertheless, we plan to implement a plan for the codevelopment of a novel ethical oversight framework for DSHR in Nigeria, a typical LMIC African country, that is built on the insights generated from this research [32-34]. The current study’s outcome will also inform global ethical discourse on DSHR and the development of ethical oversight for DSHR in different parts of the world.

## Methods

### Theoretical Framework

**Figure 1:**
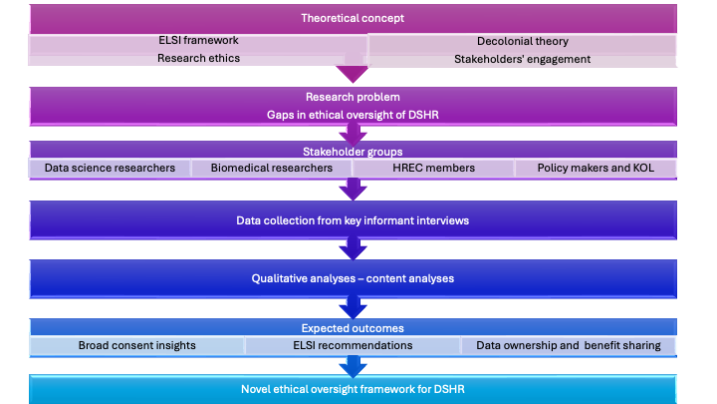
Study design and conceptual flow. DSHR: data science health research; ELSI: ethical, legal, and social implications; HREC: Health Research Ethics Committee; KOL: key opinion leader.

We engage multiple theoretical frameworks for this study (Figure 1). This includes classical research ethics principles, ELSI framework, decolonial and postcolonial theory, and stakeholder engagement theory. The classical ethical theory rests on principles articulated in the Belmont report and expanded in subsequent writings [35-37]. This includes respect for persons, beneficence and nonmaleficence, and justice. The ELSI framework, which was originally developed for genomics, provides a systematic approach to identifying the broader societal implications of emerging technologies in research [38,39]. It encompasses ethical, legal, and social (ELS) dimensions. Our analyses embrace decolonial and postcolonial perspectives, which illuminate data colonialism, extractive research, epistemic justice, and structural power imbalances in research relationships [40,41]. Lastly, we use stakeholder engagement theory, which posits that meaningful participation of all parties with interests in or affected by research leads to more legitimate, acceptable, and effective outcomes. In this study, we integrate these theoretical frameworks into a mutually reinforcing approach that honors universal ethical principles while remaining contextually appropriate; addressing novel technological challenges while learning from established ethical precedents; confronting historical and ongoing power imbalances; and engaging all relevant stakeholders in cocreating solutions.

These theoretical frameworks directly informed our study design and implementation in (1) stakeholders’ selection wherein we included distinct groups of stakeholders who contribute unique experiences and expertise, (2) the constructs that were used to organize the research questions, (3) analysis approach which uses deductive and inductive coding and allows for theory driven analyses, and (4) expected outcomes. The frameworks support the exploration of our study’s questions, including knowledge and awareness of existing ethical frameworks and legal instruments, current ethical oversight, a novel ethical framework for DSHR, equity, and justice. The strengths of our frameworks include their comprehensive coverage of the ethics of DSHR, contextual sensitivity, practical applicability, and flexibility.

### Study Design

We will use the Key Informant Interview (KII) qualitative research method for this study.

### Setting

This study will be conducted in Nigeria, the most populous (237 million) and one of the largest economies in Africa, with a purchasing power parity gross domestic product of US $2.6 trillion in 2025 [42].

### Participants

In this study, we will recruit individuals from 4 major stakeholder groups across Nigeria to participate in KII. These groups are:

1. Health researchers who are using data science research methods for their projects in Nigeria.
2. Members of Health Research Ethics Committees (HREC) that have reviewed DSHR proposals.
3. Data scientists who are conducting DSHR.
4. Policymakers, funders, and key opinion leaders (KOL) in DSHR in Nigeria.

We used lists of prime or subcontract awardees in the National Institutes of Health’s Harnessing Data Science for Health Discovery and Innovation in Africa (DS-I Africa) program in Nigeria, obtained from the DS-I Africa Program Officers, to identify researchers involved in data science projects and members of the ethics committees in the institutions hosting DS-I Africa projects in Nigeria. Further, we searched databases such as PubMed, Scopus, Web of Science, etc, for researchers who have published papers on DSHR in Nigeria. We contacted officials of the Nigerian Ministries of Health, Justice, Communication, Science and Technology, and Education to identify policymakers responsible for health research and data science in the public sector in Nigeria. Given the active and successful private data science sector in Nigeria, we also contacted relevant trade organizations and invited their members to participate in this study. We identified other KOLs on data science, health, and research in Nigeria through publicly available information and invited them to participate in this study [38]. We used multiple strategies to invite participants, including phone calls, personal contacts, and emails.

### Design of KII Guide

We developed a semistructured KII guide organized by constructs based on common perceptions and published literature on data science, health research, ethics, and protection of individuals whose data may be used in DSHR (Multimedia Appendix 1). The KII guides included the following questions:

1. Awareness and knowledge of policies and laws related to data science in Nigeria.
2. The ethical oversight of the design and implementation of research projects in Nigeria.
3. What and how the ELS issues associated with data science are considered in the development of institutional and national policies for ethical research in Nigeria.
4. Awareness and knowledge of the ELS issues related to DSHR in Nigeria.
5. Experience with addressing the ELS issues associated with data science research in Nigeria.
6. Description of the processes and organizations relevant to the oversight of DSHR in Nigeria. What are the benefits, limits, and gaps in the policies and organizations?
7. The components of an ideal oversight framework and policies for DSHR.
8. The roles of researchers, patients, research participants, groups, and communities in the development of ethical oversight of DSHR.
9. The impact of DSHR on research ethics and policy in Nigeria and vice versa.
10. The roles of Nigerian laws in researchers’ decisions about the processes and practices of data science research.
11. The policies that enhance equity and justice in the deployment of data science tools for health research across different socioeconomic groups in Nigeria.
12. How Nigeria can implement adequate regulations while facilitating collaborations and partnerships that prioritize health research goals and innovations in Nigeria.

We also collected demographic data and information on past training in research ethics and data science from the participants.

### Sample Size, Data Collection, Management, and Analyses

We will interview 65 participants. Based on the principles for computing sample size for qualitative studies, data saturation is typically reached with 5 to 10 participants. So, we plan to recruit 5 to 10 participants in each stakeholders’ category [28,29]. Though some participants may represent multiple categories of stakeholders, we will ensure a minimum of 5 interviewees per category, as shown in Table 1.

**Table 1:** Categories and number of participants in each stakeholder group, Nigeria 2024.

Participants’ categories		Participants, n	
**Age (years)**			
	<40		5
	40-60		5
	>60		5
**Sex**			
	Male		5
	Female		5
	Others		5
**Discipline**			
	Data science		5
	Health		5
	Biology		5
	Computer science		5
	GISs^a^		5
	Research ethics		5
	Policymaker		5
	Others		5
**Domain**			
	Commercial		5
	NIH^b^ and other agencies		5
	Foundation-sponsored groups		5
	Local government-funded groups		5
	Private sector-funded groups		5
	Consortia		5
	Collaborations		5
Researchers involved in data science projects in Nigeria		5	
Members of the ethics committees of institutions hosting DS-I Africa^c^ projects in Nigeria		5	
Officials of Ministries of Health, Justice, Communication, Science and Technology, and Education in Nigeria		5	
Key stakeholders in the Nigerian data science private sector		5	

^a^GIS: geographic information system.

^b^NIH: United States National Institutes of Health.

^c^DS-I Africa: Harnessing Data Science for Health Discovery and Innovation in Africa.

### Data Collection

The KIIs would be conducted by a research associate assisted by a notetaker who would take notes and record participants’ nonverbal cues, which would be used to supplement the audio recordings. Interviews would be conducted in person and virtually using zoom (Zoom Communications, Inc). Each interview would last 60 to 90 minutes, and we would use the KII guide to guide the interviews. All interviews will be recorded, and we will transcribe the recordings to generate transcripts. We will work with KII participants to clarify their comments and correct the information they provide as required. Before conducting the KIIs, participants would be provided with an information sheet outlining this study’s objectives, confidentiality measures, and their rights as participants.

### Qualitative Data Analyses

We will conduct content analysis using a combination of deductive and inductive processes with manual and computer-assisted data analyses using ATLAS.ti (version 7.5.2; ATLAS.ti Scientific Software Development GmbH) software. We will create a priori codes based on our research objective, as a general framework, while allowing for emergent concepts from the discussions. For each code, we will write a description in the code manager to provide contextual information. The coding would be an iterative process, with the first level of coding generating initial codes that would be evaluated before a second and third level of coding. We will group codes into families according to shared themes and create semantic networks representing hierarchical relationships between concepts after a review of the codes. We will analyze each code to identify emerging opinions and themes for the research objective. The reflective and thematic memos that we keep during the coding process would be used to provide contextual information. We will use query tools and Boolean operators to interrogate the codes and obtain the results that will be published using the COREQ-32 (Consolidated Criteria for Reporting Qualitative Research) [42]. We will use simple descriptive statistical analyses for the quantitative data associated with these qualitative data.

### Ethical Considerations

This study was approved by the National Health Research Ethics Committee of Nigeria (NHREC/01/01/2007-24/12/2024D) and the Institutional Review Board of the University of Maryland, Baltimore (HP-00102012). It was conducted according to the guidelines in the Nigerian National Code for Health Research. Informed consent was obtained from all the participants whom we interviewed. Participants were compensated with US $2 for attending the interviews. All audio recordings and transcripts were deidentified and stored in password-protected computers.

## Results

### Reasons for Nonresponse Among Participants Invited for the KII

Some 25% (n=22) of the individuals who were invited were unable to participate in the KIIs. Table 2 shows the main reasons for the inability to participate. Most (13/22, 59%) did not respond to our invitation, 27% (6/22) declined to participate, while the remaining 14% (3/22) were unable to participate due to logistical or scheduling constraints, and bureaucratic delays. Among those who did not respond to our invitation to participate in this study, data science researchers were the largest group (5/13, 39%), followed by policymakers (3/13, 23%), HREC members (3/13, 23%), and biomedical researchers (2/13, 15%). Of the 6 participants who declined to take part in the KII, most (5/6, 83%) were data science health researchers, while the remaining 1 (1/6, 17%) person was a biomedical researcher. The 3 participants who were unable to participate due to scheduling difficulties and bureaucratic delays were 1 policymaker, 1 data science health researcher, and 1 biomedical researcher.

**Table 2:** Response rate of participants invited for study, Nigeria 2024.

Reasons			Values, n (%)
**Nonresponse**			13 (100)
	Policymaker	3 (23)	
	Data science health researcher	5 (39)	
	Health Research Ethics Committee	3 (23)	
	Health data scientist	2 (15)	
**Declined**			6 (100)
	Data science health researcher	5 (83)	
	Health data scientist	1 (17)	
**Others**			3 (100)
	Policymaker	1 (33)	
	Data science health researcher	1 (33)	
	Health data scientist	1 (33)	

### Analysis of Quantitative Data

Table 3 shows the baseline characteristics of the 65 stakeholders who participated in this study. Their mean age was 47.9 (SD 7.9) years. Most (50/65, 77%) were male, while 23% (15/65) were female. More than half (38/65, 57%) of the participants had doctoral degrees, 25% (16/65) had master’s degrees, and 19% (11/65) had only a first degree. Some 39% (25/65) of the participants identified as data science health researchers, 26% (17/65) were biomedical researchers, 19% (12/65) were members of HREC, and 17% (11/65) were policymakers. Their affiliations were mainly with academia (45/65, 69%), government organizations (26/65, 40%), nongovernmental organizations (7/65, 11%), commercial entities (2/65, 3%), and teaching hospitals (18/65, 28%). Most (50/65, 77%) reported having received training in research ethics. Of these, 59% (38/65) had completed short-term training, 12% (8/65) long-term training, and 6% (4/65) medium-duration training. Most of the participants (57/65, 88%) had not received any training in the ethics of DSHR, while 12% (8/65) had received such training. Among those who had received ethics of DSHR training, 88% (7/8) completed short-duration training, while 13% (1/8) had medium-duration training. Most (60/65, 92%) of the participants agreed that there is a need for specific training in the ethics of DSHR.

**Table 3:** Baseline characteristics of study participants, Nigeria 2024.

Variables				Participants, n (%)
**Sex**				
		Male	50 (77)	
		Female	15 (23)	
**Education**				
		First degree	11 (17)	
		Masters	16 (25)	
		PhD	38 (59)	
**Category**				
	Policymakers		11 (17)	
	Data science health researchers		25 (39)	
	Health Research Ethics Committee		12 (19)	
	Health data scientist		17 (26)	
**Role within the HREC^a^**				
	Chairperson		5 (42)	
	Member		7 (58)	
**Group within HREC**				
	Scientist		11 (92)	
	Nonscientist		1 (8)	
**Data science health researcher**				
	Directly use DSM^b^		15 (60)	
	Work with others who use DSM		19 (76)	
	Do not use DSM		0 (0)	
	Others		1 (4)	
**Current data science role**				
	Administrative or managerial		2 (12)	
	Project lead or principal investigator		7 (41)	
	Data or ML^c^ engineering		8 (47)	
	Data scientist		14 (82)	
	Statistician or bioinformatician		1 (6)	
	Data architect		2 (12)	
	Data analyst		7 (41)	
	No direct data science role		0 (0)	
	Other		3 (18)	
**Affiliation**				
	Academia		45 (69)	
	Government		26 (40)	
	NGO^d^		7 (11)	
	Commercial		2 (3)	
	Teaching hospital		18 (28)	
**Level of training received in research ethics**				
	Short (<2 months)		38 (59)	
	Medium (at least 2 months)		4 (6)	
	Long (>2 months)		8 (12)	
	None		15 (23)	
**Level of training received in the ethics of DSHR^e^**				
	Short (<2 months)		7 (11)	
	Medium (at least 2 months)		1 (2)	
	Long (>2 months)		0 (0)	
	None		57 (88)	
**Do you think there should be specific training in the ethics of DSHR?**				
	Yes		60 (92)	
	No		5 (8)	

^a^HREC: Health Research Ethics Committee.

^b^DSM: data science methods.

^c^ML: machine learning.

^d^NGO: nongovernmental organization.

^e^DSHR: data science health research.

### Study Timeline

As of January 2025, the interview transcripts have been generated and checking completed, with qualitative analysis scheduled for completion by March 2025 and completion of primary manuscripts by the end of 2025.

## Discussion

### Principal Findings

This is the first comprehensive, qualitative investigation of the perspectives of multiple stakeholders on the ethical oversight for DSHR in Nigeria, a typical African LMIC. Of the 87 individuals invited, 22 (25.3%) were unable to participate, with 59% (13/22) not responding to invitations. Data science researchers comprised the largest group among nonresponders (5/13, 39%), which may reflect time constraints, research fatigue, or discomfort with ethics-focused discussions. This pattern warrants attention in future engagement efforts and suggests the need for sustained iterative approaches to stakeholder consultation in DSHR ethics development. The demographic profile of our participants reflects the current landscape of DSHR engagement in Nigeria, with most participants (50/65, 77%) being male, over half holding doctoral degrees (38/65, 57%), and the majority affiliated with academic institutions (45/65, 69%) and government organizations (26/65, 40%).

A striking finding was the substantial lack of specialized training in DSHR ethics, with only 12% (8/65) having received any training specific to the ethics of DSHR, and this was predominantly short-duration training. This deficit is particularly concerning given that 92% (60/65) of participants acknowledged the urgent need for specialized DSHR ethics training. This finding aligns with recent observations about the lag between technological advancement, such as DSHR, and the development of appropriate ethical oversight mechanisms [30,43]. The high proportion of participants who recognize the need for training of stakeholders in the ethical aspects of DSHR suggests receptiveness to capacity-building initiatives and readiness for meaningful engagement in developing contextually appropriate ethical frameworks.

### Comparison to Prior Work

Our study contributes to the growing body of literature examining ethical governance of DSHR in Africa. Recent work emphasized that DSHR in Africa poses unique challenges, including blurred consent boundaries, reidentification risks, algorithmic biases, and transnational commercial exploitation [30]. Our multistakeholder approach directly addresses previous recommendations for engaging diverse perspectives in developing novel ethical frameworks suitable for resource-limited settings. The methodology we used in this study aligns with established best practices in research ethics policy development, similar to the 7Ps Framework for stakeholder identification in Patient-Centered Outcomes Research, emphasizing the importance of including patients, providers, purchasers, payers, policymakers, product makers, and principal investigators [44]. Our study adapted the 7Ps framework to the DSHR context in Nigeria, ensuring representation from researchers (both data science and biomedical), ethics committee members, research sponsors, and policymakers. Recent work has demonstrated that effective stakeholder engagement requires clarifying objectives, embedding engagement in research frameworks, and fostering collaborative relationships, which are the principles that we operationalized through our semistructured interview approach [45]. The training deficit we identified resonates with broader assessments of research ethics capacity in Africa. While substantial progress in building general research ethics capacity has been made, most of these assessments of ethics capacity occurred before the rapid expansion of DSHR and did not address the specialized competencies required for DSHR oversight [46]. Our finding that only 12.3% (8/65) of participants had received DSHR-specific ethics training highlights a critical gap that has emerged recently and requires focused intervention.

On broad consent, which we anticipate would emerge as a major theme when we complete our qualitative analysis, a recent comprehensive review highlighted that while broad consent enables data sharing for secondary research, concerns remain about whether it adequately protects participants’ autonomy and community interests in LMICs [47]. Stakeholders’ preferences have also been found to be highly varied [48]. Our study’s inclusion of multiple stakeholders, including policymakers who shape consent frameworks, positions us to contribute nuanced insights about the applications of broad consent to DSHR in Nigerian contexts. Algorithmic bias emerged as a critical ELSI concern in our literature review and is likely to feature prominently in our analysis. Recent work in this area demonstrated that algorithmic bias poses substantial challenges in Africa, particularly regarding fairness and integrity of AI applications, with the potential to perpetuate data colonization if large multinational corporations impose solutions without local innovation [49]. It may constitute a “silent threat to equity” because equity must be engineered as a foundational design principle in DSHR rather than retrofitted into algorithms, data, or analyses [50]. These observations underscore the urgency of developing ethical frameworks that explicitly address algorithmic bias before DSHR tools are widely deployed in Nigerian health care contexts.

The challenge of data sharing and ownership in African health research has been well-documented. Data sharing between African and international health researchers is influenced by ethical, legal, social, institutional, and governmental factors, and freighted with concerns about predatory inclusion and inadequate benefit sharing [51]. While Open Science principles are increasingly applied by African researchers, the “inverse care law” and digital data poverty risks amplification of extractive data practices [51]. By focusing on stakeholder perspectives regarding data ownership and benefits sharing directly, we will be able to address these concerns within the specific context of DSHR in Nigeria. Recent work on data protection legislation across Africa identified considerable variation in requirements for cross-border data transfers and secondary data use, with enumeration of several proposals to improve data sharing, including standardized modules for safe data flows, trusted data environments, dynamic consent mechanisms, and codes of conduct for secondary data use [52]. We anticipate that similar themes would emerge from our stakeholder consultations. By including policymakers and KOLs in our stakeholders’ group, we recognize that ethical oversight of DSHR requires engagement beyond traditional research participant communities to include those shaping broader societal understanding and governance of data science.

LMICs must develop appropriate guidelines for the ethical oversight of DSHR. This is particularly urgent in countries such as Nigeria because of the need to protect data donors, donor communities, and local researchers. At the same time, LMICs need to promote DSHR to take advantage of this powerful new resource and use it to solve many hitherto intractable health problems. LMICs need to contribute data to global databases to alleviate data poverty, which contributes to algorithmic and other biases, and the limited effectiveness of data science tools in their environment. LMICs need to develop the right infrastructure, manpower, training, and oversight mechanisms for meaningful participation of their researchers and communities in global DSHR [53].

### Strength and Future Directions

Our study has several conceptual and methodological strengths that enhance its validity and potential impacts. The multistakeholder approach ensures that we capture diverse perspectives, and this responds to prevalent power asymmetry in policy development. We also operationalize the principle of epistemic humility and equitable participation [54]. This inclusive design increases the likelihood that recommendations arising from our study will be contextually appropriate, broadly acceptable, and implementable within Nigeria’s research ecosystem. The hybrid deductive-inductive content analysis approach provides methodological rigor while remaining open to emergent themes not captured in existing literature. We will use an iterative 3-level coding process with thematic families and semantic networks that enable identification of complex relationships between concepts crucial for understanding how multiple ELSIs intersect in DSHR contexts. The commitment to reporting findings according to COREQ-32 criteria enhances the transparency and reproducibility of our study, aligning with international standards for qualitative health research reporting. Our focus on Nigeria, a large and populous country with a rapidly expanding data science use environment and well-established research ethics infrastructure, yet with gaps reminiscent of other LMICs, ensures that our findings would be generalizable to other LMICs.

Our findings would serve as resource materials for training in the ethics of DSHR and creation of standard operating procedures for ethics committees addressing DSHR proposals, particularly in helping to focus on consent frameworks for secondary data use, algorithmic bias assessment, and cross-border data sharing governance. Future studies should examine the implementation and effectiveness of our recommendations and conduct longitudinal assessments during the evolution of the ethical oversight of DSHR in Nigerian institutions. Comparative studies examining stakeholder perspectives across multiple African countries would test the generalizability of findings and potentially inform regional harmonization efforts for DSHR governance. Additionally, future work should explore mechanisms for dynamic and ongoing stakeholder engagement in DSHR ethics as the field continues to evolve. The rapid pace of technological change in data science means that static ethical frameworks quickly become outdated. Establishing communities of practice that bring together researchers, ethicists, policymakers, and community representatives for regular deliberation about emerging DSHR ethics challenges would create adaptive governance capacity.

Our study has several limitations. The 25% nonparticipation rate, consisting mainly of data scientists, introduces potential selection bias. This limitation is partially mitigated by our large sample size, which still included a lot of data scientists. Nevertheless, our findings should be interpreted with awareness that the most reluctant or time-constrained data scientists may hold perspectives different from those who participated. Second, our study’s cross-sectional design captures stakeholder perspectives at a single time point at an early stage in the implementation of DSHR in Africa. Perspectives may evolve as stakeholders gain more experience with DSHR, with changes in DSHR technologies and methods, and as the ethical challenges of DSHR become more apparent through real-world applications. Our study partially addresses this limitation through its planned dissemination and engagement activities, which will enable longitudinal tracking of the evolution of perspective on ethical oversight of DSHR in future research. The documentation of substantial variation in participants’ training and experience levels provides some insight into how perspectives might evolve with increased engagement. Third, our interview-based methodology, while providing rich qualitative data, does not capture quantitative measures of stakeholder agreement or priorities. Future phases of framework development would benefit from mixed methods approaches, perhaps using Delphi techniques to achieve consensus on key principles and priorities. Such approaches would complement the qualitative insights from this study with quantitative data on stakeholder priorities.

Finally, our study occurs within a specific regulatory and technological context that is rapidly evolving. Nigeria’s data protection legislation, the Nigeria Data Protection Act 2023, was recently enacted and is still being implemented, meaning stakeholders’ understanding of its implications may be incomplete. Similarly, rapid advances in AI and machine learning mean that the DSHR landscape stakeholders described may quickly change. These temporal limitations necessitate ongoing monitoring and periodic reassessment of ethical frameworks developed from our study findings. More research and community consultations to validate our findings would be required.

### Dissemination of Findings

We are committed to the widespread dissemination of our findings. We will disseminate our findings to all stakeholders in the research ecosystem through submission of a report to members of national and institutional HRECs, presentations at research conferences and meetings, and in peer-reviewed publications. We will communicate our results directly to the volunteers who participated in our study, DS-I Africa principal investigators, and project sponsors. We will work with the sponsors to advocate for our findings using personal contacts, webinars, conferences, and workshops so they can influence DSHR stakeholders in Nigeria and other African countries.

### Conclusions

This is the first study that we are aware of that will generate stakeholder-informed recommendations for ethical oversight of DSHR within resource-limited settings. Participants will contribute insights on how to address the applications and scope of broad consent for data use, the ELS challenges, data ownership and data sovereignty, benefit-sharing, and donor protection in DSHR. The findings will inform the global DSHR and research ethics communities and guide them on the implementation of contextually appropriate oversight mechanisms that promote equitable partnerships, co-ownership, and tiered data governance while protecting the rights of data donors and advancing global health research innovation.

## Appendix

Multimedia Appendix 1 Key Informant Interview Guides.

Multimedia Appendix 2 Peer review report from the Center for Scientific Review Special Emphasis Panel RFA-RM-20-017: Harnessing Data Science for Health Discovery and Innovation in Africa – Ethical, Legal and Social Implications Research (National Institutes of Health, USA).

## Abbreviations

AI: artificial intelligence
COREQ-32: Consolidated Criteria for Reporting Qualitative Research
DSHR: data science health research
DS-I Africa: Harnessing Data Science for Health Discovery and Innovation in Africa
ELS: ethical, legal, and social
ELSI: ethical, legal, and social implication
HREC: Health Research Ethics Committee
KOL: key opinion leader
KII: Key Informant Interview
LMIC: low- and middle-income country

## Data Availability

Data from this study will be available in a publicly accessible data repository according to the National Institutes of Health Data Sharing Guidelines and will be provided by the corresponding author upon receipt of reasonable requests.
