# Supplementary material for: Knowledge and Recommendations of Stakeholders Regarding Ethical Oversight of Data Science Health Research: Protocol for a Qualitative Study
Source: JMIR Res Protoc. 2025 Dec 18;14:e78557. doi: 10.2196/78557 (PMC12757715; doi:10.2196/78557)
Supplement: Multimedia Appendix 1 [file resprot_v14i1e78557_app1.docx]

**Information sheet for Key Informant Interviews in the Bridging Gaps in the ELSI of Data Science Research (BridgELSI) Project**

This Information Sheet (IS) serves to provide you with information about the Bridging Gaps in the ELSI of Data Science Research (BridgELSI) Project and the objectives of the Key Informant Interviews that are part of the project.

The aim of the BridgELSI Project is to study the ethical, legal, and social implications (ELSI) of the use of data science in health research in Nigeria. Data Science is defined as “the use of quantitative and analytical approaches, processes, and systems developed and used to extract knowledge and insights from increasingly large and/or complex sets of data”. It is a method of conducting research that uses large amounts of information collected with or without permission, e.g., by collecting data on the places people visit on the Internet and the topics they search for to predict diseases that people who visit similar places or conduct similar searches may have in future. These types of research use different methods such as online activities, phone location data, items purchased in the market or direct questions from individuals, communities, or organizations to generate novel insights and knowledge by linkages and analyses.

Conducting these types of research uses huge amounts of data which can come from combining the data of millions of people in different parts of the world. The data is put into high performance computers with power that is equivalent to millions of laptops, and researchers use very complex mathematical methods to analyze the data. Because of the huge amount of data involved, use of extremely powerful computers and complex mathematics, many terms including artificial intelligence or machine learning are used to describe these types of research.

The novelty and complexity of these methods when applied to health research raise many new questions about how to oversee these types of research and ensure that individuals and communities whose data is used for are protected from needless harm. Our research team is interested in how to provide ethical oversight for these data science health research in Nigeria.

At this time, we want to know what you currently know, believe, and practice about these types of research, not what you expect, or believe should happen. We want you to focus on the methods we described and their applications in health research area only. We hope that this IS has provided you with enough information to enable you engage in serious and in-depth discussions with us during the interview. Let us know if anything is not clear or if you require more information.
