# Supplementary material for: Knowledge and Recommendations of Stakeholders Regarding Ethical Oversight of Data Science Health Research: Protocol for a Qualitative Study
Source: JMIR Res Protoc. 2025 Dec 18;14:e78557. doi: 10.2196/78557 (PMC12757715; doi:10.2196/78557)
Supplement: Multimedia Appendix 2 [file resprot_v14i1e78557_app2.pdf]

1U01MH127693-01 Ogundiran, Temidayo

BUDGETARY OVERLAP

RESUME AND SUMMARY OF DISCUSSION: This application proposes to improve data science health research in Nigeria through a combination of evaluation of current frameworks, engaging stakeholders, and providing training for researchers and ethics committee members. Since foundational work in the ethical oversight of data science health research in Nigeria is lacking, and there is a clear and demonstrated need for robust governance, this application fills a gap by engaging stakeholders to ultimately develop a governance framework for the ELSI of data science health research. The panel agreed that the investigative team is excellent, with strong ethics training and relevant experience translating this type of work into high-impact policy and training. Methodological strengths of the application include the longitudinal nature of the KAP surveys, stakeholder engagement, and a flexible approach that will allow for adapting to changing research needs. There was some disagreement on the committee over the training component in the final aim; while some panel members did not view the training component as specific to the ELSI of data science research, others believed that it represents an innovative component of the application with implications for dissemination and capacity-building. The panel identified several relatively minor weaknesses, including potentially limited generalizability beyond Nigeria, a lack of clear outputs for several of the aims, and a lack of justification for the inclusion of members of the general public on the KAP survey. Overall, the panel believed the application has the potential for a high impact on African ELSI research.

DESCRIPTION (provided by applicant): Data science is poised to impact scientific research, innovation, discovery and healthcare in sub-Saharan Africa (SSA) because of the rapid growth of infrastructure such as cell phones and computers, and the availability of technologies like Artificial Intelligence. These methods and resources present huge opportunities to leapfrog current research, public health and clinical care in Africa by utilizing data science to address the huge burden of communicable and non-communicable diseases in SSA. Despite these promises however, there are substantial concerns about the ethical, legal and social implications (ELSI) of data science research in SSA. These concerns arise from the use of conventional and unconventional data; the methods for generating, manipulating, storing, sharing and utilizing data in data science; the limitations of current informed consent models in these scenarios and opportunities for novel strategies for legal oversight of the ELSI of data science research in Nigeria. In this collaborative project between the Center for Bioethics and Research (CBR), Nigeria, George Washington University, DC and University of Maryland School of Medicine (UMSOM), we will evaluate current legal instruments, guidelines and frameworks, and their implementation, and use these to develop new and innovative governance frameworks to support data science health research in Nigeria. We will also implement mixed research methods to prospectively evaluate the knowledge, attitude and practices (KAP) of data scientists and ethics committees to current and emerging ELSI of data science research in Nigeria. Given the novelty of data science in Nigeria, we will implement general and specific, short and medium-term training in ethics of data science research in Nigeria for data science researchers and an introduction to data science for members of ethics committees reviewing data science research projects.

PUBLIC HEALTH RELEVANCE: The Bridging Gaps in the ELSI of Data Science Research (BridgELSI) Project is a collaborative research ethics study and training project that is focused on bridging the gaps in current governance framework on ethical oversight of data science health research by engaging key stakeholders to evaluate and co-develop novel governance framework for the Ethical Legal and Social Implications (ELSI) of data science health research in Nigeria. The project will implement short and medium-term training on ethics of data science for data scientists, ethicists and ethics committees, and strengthen the ethical working environment for data science health research in
